# Deficiency of Adenosine Deaminase 2 in Adult Siblings: Many Years of a Misdiagnosed Disease With Severe Consequences

**DOI:** 10.3389/fimmu.2018.01361

**Published:** 2018-06-14

**Authors:** Jason Michael Springer, Selina A. Gierer, Hong Jiang, David Kleiner, Natalie Deuitch, Amanda K. Ombrello, Peter C. Grayson, Ivona Aksentijevich

**Affiliations:** ^1^Division of Allergy, Clinical Immunology and Rheumatology, Kansas University Medical Center, Kansas City, MO, United States; ^2^Laboratory of Pathology, National Cancer Institute, National Institutes of Health, Bethesda, MD, United States; ^3^National Human Genome Research Institute, National Institute of Health, Bethesda, MD, United States; ^4^National Institute of Arthritis and Musculoskeletal and Skin Diseases, National Institute of Health, Bethesda, MD, United States

**Keywords:** adenosine deaminase 2 deficiency, adenosine deaminase 2, vasculitis, nodular regenerative hyperplasia, stroke, polyarteritis nodosa

## Abstract

**Objective:**

Describe the clinical characteristics and histopathology findings in a family with two siblings affected with deficiency of adenosine deaminase 2 (DADA2). Both patients presented in childhood with polyarthritis and developed significant neurological and gastrointestinal features of DADA2 in ear, including variable degrees of immunologic and hematologic manifestations.

**Methods:**

Adenosine Deaminase 2 (ADA2; also known as *cat eye syndrome chromosome region, candidate 1 gene; CECR1)* exon sequencing and serum ADA2 levels were performed to confirm the diagnosis of DADA2. Comparison of serum adenosine deaminase 2 levels was made to DADA2 patients, carriers, and healthy controls in Patient 2. Autopsy specimens from brain and liver tissues were submitted for analysis.

**Results:**

Both patients were found to carry a previously reported rare intronic missense mutation predicted to affect the transcript splicing (c.973-2A > G; rs139750129) and an unreported missense mutation p.Val458Asp (c.1373T > A; V458D). Both brothers started therapy with a tumor necrosis factor inhibitor following the molecular diagnosis of DADA2 with good response and were eventually tapered off prednisone. However, Patient 1 died 18 months later due to complications of end-stage liver disease. His autopsy showed evidence for nodular hyperplasia of the liver often seen in common variable immunodeficiency (CVID) and numerous small, old infarcts throughout the brain that had not been demonstrated on prior MRI/MRA imaging.

**Conclusion:**

These cases emphasize the importance of recognition of DADA2 in adults, compare CNS imaging modalities to pathologic findings and suggest similarities in liver pathology between DADA2 and CVID. MRI may not be most sensitive method to identify small subcortical infarcts in patients suspected to have DADA2.

## Introduction

Deficiency of adenosine deaminase 2 (DADA2) is a recently described monogenic autoinflammatory disease that can mimic polyarteritis nodosa (PAN). DADA2 is associated with bi-allelic recessively inherited loss-of-function mutations in the cat eye syndrome ADA2 (1, 2). The majority of the reported cases have been diagnosed in children. Herein, we report cases of two brothers who presented with DADA2-associated features in early-adulthood, including progressive neurological impairments, gastrointestinal manifestations, immune dysregulation, and a severe portal hypertension. Initiation of treatment with TNF inhibitors soon after the molecular confirmation of DADA2 resulted in significant ameliorations in their condition. Despite the improvement, one sibling had irreversible end-stage liver disease resulting in death at the age of 42. Here, we report the brain and liver autopsy findings in DADA2.

## Patients and Methods

### Case 1

A 40-year-old Caucasian male, with a prior diagnosis of PAN, presented in January 2016 with large volume hematemesis from esophageal varices related to portal hypertension. Throughout his childhood he had chronic arthralgia and swelling of his ankles diagnosed as juvenile idiopathic arthritis. By age 4, he manifested developmental delays, including difficulty with ambulation. At the age of 20, he developed recurrent ischemic strokes causing persistent dysarthria, ataxia, and weakness. During that time, he developed recurrent violaceous subcutaneous lesions that would ulcerate, but were responsive to glucocorticoids. He was diagnosed with PAN based on skin biopsies. He required a long-term maintenance dose of prednisone 7.5 mg daily, as reduction would led to recurrent skin lesions. In childhood, he developed poorly explained visual loss without evidence of inflammatory eye disease on physical exam. His visual acuity had been stable for the last couple years prior to presentation. His history was also remarkable for recurrent mouth and genital ulcers, which were also responsive to glucocorticoids. He had lymphopenia (absolute lymphocytes count 400/μl [1000-4800], CD3 422/μl [600-2990], CD4 370/μl [440-2160], CD8 49/μl [120-1320], CD19 11/μl [100-700]), yet normal lymphocyte proliferation studies to mitogens. Additionally, he had hypogammaglobulinemia (IgG 383 mg/dl [762-1488], IgA 46 [70-390], IgM <20 [38-328]), but had protective antibody levels to both tetanus and pneumococcus. There was no history of recurrent infections.

### Case 2

A 43-year-old Caucasian male, brother to Patient 1, had a similar presentation. At 5 years of age, he was diagnosed with juvenile idiopathic arthritis after presenting with polyarthritis. At the age of 8, he was diagnosed with stage IV Hodgkin’s lymphoma (records were not available). He was initially treated with radiation therapy alone, but later transitioned to chemotherapy [initially with MOPP (mechlorethamine, vincristine, procarbazine, and prednisone) and later converted to ABVD (doxorubicin, bleomycin, vinblastine, and dacarbazine)] with remission achieved 1 year later. At age 24, he developed sudden onset left-sided persistent numbness followed by bilateral sensorineural hearing loss with near complete left-sided deafness. At age 26, he presented with bright red blood in his stool, which required resection of 6 inches of small intestines for reasons that were unclear. By age 29, he developed heart failure and pulmonary hypertension presumed secondary to ABVD treatment. His history was also significant for superior vena cava occlusion, presumed secondary to a port catheter, and esophageal varices. Immunologic evaluation revealed normal immunoglobulins, lymphocyte proliferation to mitogens, and lymphocyte subsets except for a slightly low CD8 count (83/μl). He had protective antibody levels to tetanus.

Based on these presentations, there was a high suspicion for DADA2. Written informed consent was obtained for each patient, as well as their parents for genetic testing, and for the publication of this case report. *ADA2* exon sequencing was performed on both patients and their unaffected parents as previously described (1). Adenosine deaminase 2 levels were tested only in Patient 2 with comparisons made to DADA2 patients, adult carriers for *ADA2* mutations and adult healthy controls.

## Results

**Figure 1:**
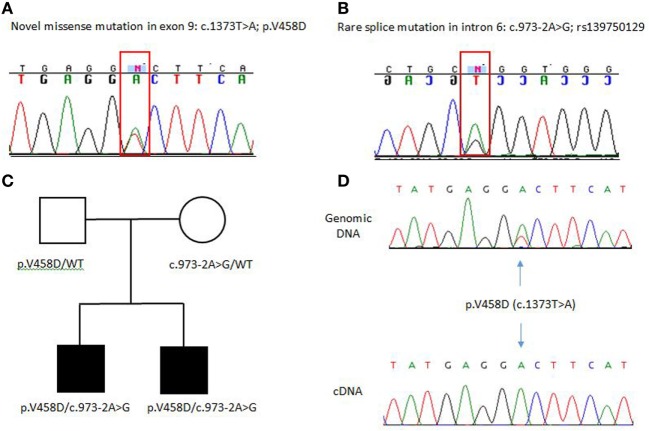
**(A,B)** Electropherograms of ADA2 pathogenic mutations identified in the family. **(C)** Family pedigree, both unaffected parents are carriers of these mutations. WT indicated wild-type or non-mutated ADA2 allele. **(D)** cDNA sequencing shows that the p.V458D mutation appears heterozygous at the genomic DNA and as a homozygous mutant allele when transcribed in cDNA indicating null expression of the second allele due to the c.973-2A>G mutation.

**Figure 2:**
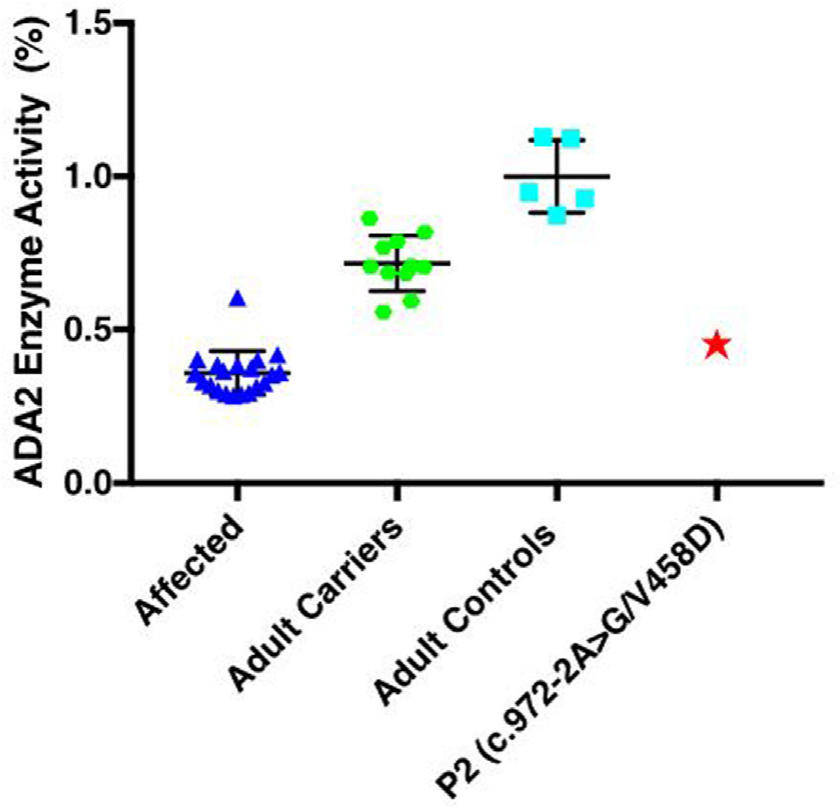
Serum ADA2 enzyme activity and cDNA sequencing to confirm pathogenic mutations: enzymatic activity (%) was significantly lower in the affected Patient 2 when compared to age-matched controls (*n* = 5) and heterozygous carriers (*n* = 11), but similar to other patients with DADA2 (*n* = 20). Samples were not available for patient.

Both brothers were found to have two disease-causing mutations: a rare intronic missense mutation likely affecting the splice site of exon 7 (c.973-2A > G; rs139750129) and an unreported missense mutation p.Val458Asp (c.1373T > A). The c.973-2A > G mutation has been reported in the ExAC database with a frequency of 0.0001168 in the general control population. The father was a carrier for the novel p.V458D mutation, while the mother was a carrier for the c.973-2A > G mutation. Complementary DNA (cDNA) sequencing shows that the heterozygous p.V458D mutation appears homozygous when transcribed in cDNA, indicating null expression of the second allele due to the c.972-2A > G mutation (Figure 1). We suspect the alternatively spliced transcript is unstable and degraded. In Patient 2, ADA2 levels were found to be significantly lower compared to age-matched controls and carriers (Figure 2). A sample from Patient 1 was not available for serum ADA2 testing. These findings confirmed the molecular diagnosis of DADA2 in the siblings.

**Figure 3:**
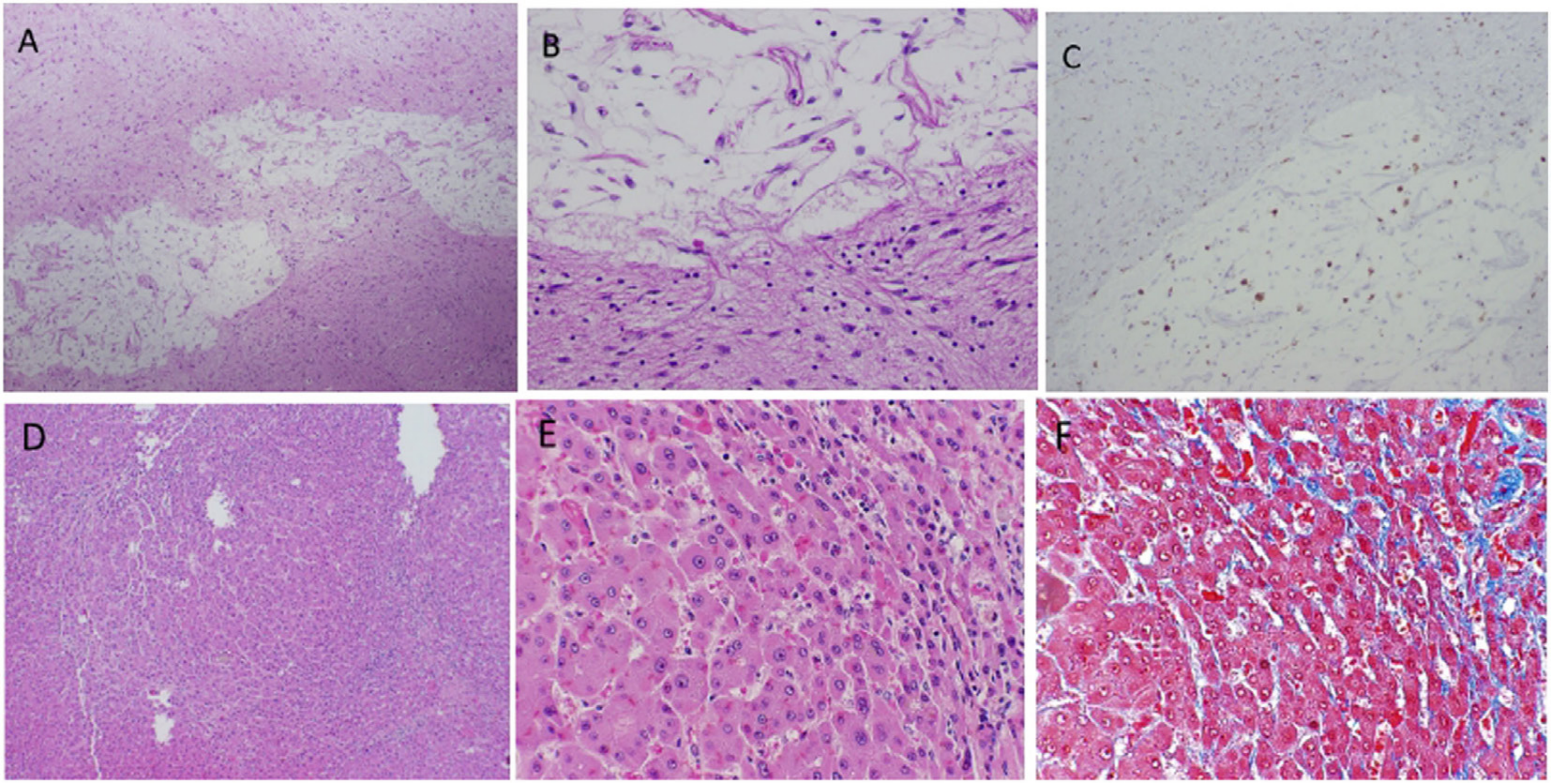
Infarcted lesion in basal ganglia **(A–C)**. **(A)** Cavity lesion with residual histiocytes and adjacent dense astrocytosis (4 × 10 magnification). **(B)** Same lesion at 20 × 10 magnification. **(C)** KP1 stain highlights histiocytes within cavity (4 × 10 magnification). Hepatic findings **(D–F)**. Nodular regenerative hyperplasia (NRH) shows in **(A)** (4 × 10) and **(B)** (20 × 10). Mild sinusoid fibrosis is seen in areas of NRH demonstrated by Trichrome Masson stain **(C)** (20 × 10).

Both brothers were initiated on etanercept 50 mg once weekly following the diagnosis of DADA2. In Patient 1, 3 months after the initiation of etanercept, there was near resolution of hypogammaglobulinemia (IgG 383 to 752 mg/dl, IgA 46 to 76 mg/dl, and IgM < 20 to 44 mg/dl), but the lymphopenia and lymphocyte subset enumeration remained unchanged. Prior to etanercept, Patient 1 was unable to taper the prednisone below a dose of 7.5 mg daily without recurrence of the vasculitic rash; however, after 6 months he was able to taper the prednisone completely. He required repeated esophageal banding procedures with progressive signs of end-stage liver disease, including hepatic encephalopathy and ascites. He passed away 18 months after his initial DADA2 diagnosis and 1 year after starting etanercept from complications related to end-stage liver disease. An autopsy revealed evidence of nodular regenerative hyperplasia (NRH) of the liver, a finding often seen in patients with disorders of hypogammaglobulinemia, particularly common variable immunodeficiency (CVID). However, the most striking finding was numerous small, old subcortical (lacunar) infarcts seen in the thalamus, basal ganglia, and brain stem sections not evident on prior MRI scans (Figure 3). In Patient 2, after 4 months on the etanercept, the C-reactive protein normalized (4.57–0.46 mg/dl) and there was no evidence of progressive neurologic features. Unfortunately, he did have progressive fatigue and myalgia. Etanercept, the soluble p75 TNFR:Fc fusion protein, was transitioned to adalimumab, a longer acting monoclonal antibody against TNF. Three months later, he demonstrated significant improvement of inflammation without progressive neurological disease.

## Discussion

One of the hallmark clinical features of DADA2 is early-onset, recurrent strokes (prevalence of 42%) with the vast majority occurring in early childhood (1). Although there are several case reports of intracerebral hemorrhage in DADA2, most patients present with ischemic strokes. Our patients developed neurological manifestations in early adulthood, illustrating that patients presenting with adult-onset neurological features and inflammation should be tested for DADA2. TNF inhibitors have been the only proven effective therapy for the prevention of DADA2-associated ischemic strokes. Hematopoietic stem cell transplantation can be curative in patients who present predominantly with bone marrow failure and/or immune dysregulation (3–5). Autopsy findings from Patient 1 demonstrated that CNS involvement can be much more widespread than what is visible on MRI. This may be related to smaller vessel involvement than what is detectable by MRI. Based on these findings we would advocate all patients with an inflammatory DADA2 phenotype and unexplained neurologic complaints to start an anti-TNF therapy despite unremarkable brain MRI/MRA findings to prevent long-term morbidity.

Bone marrow dysregulation in DADA2 includes variable degrees of anemia and bone marrow hypo/hypercellularity. Some patients have been initially diagnosed with pure red cell aplasia or Diamond–Blackfan-like anemia (6). Immunologically, patients can present with mild to profound leukopenia and antibody deficiency, in particular, low IgM. The molecular mechanisms underlying bone marrow failure remain unclear, although TNF has been described as damaging to hematopoiesis (7). Patient 1 presented with low immunoglobulins which were likely related to the DADA2, and to not his baseline immunosuppressive therapy (prednisone 7.5 mg/day). We believe the improvement in the immunoglobulins was a direct result of TNF blockade.

Interestingly, Patient 2 was diagnosed with stage IV Hodgkin’s lymphoma at a very young age. Unfortunately, limited records are available regarding the diagnosis. However, there have been multiple patients with DADA2 who have presented with lymphoproliferative-like phenotypes. Two patients have been reported to exhibit CD3^+^CD8^+^ T-cell large granular lymphocytic (T-LGL) infiltration of the bone marrow, meeting criteria for T-LGL leukemia (8). Both of these cases had somatic gain-of-function mutations in STAT3; however, this was not confined to one clonal subpopulation or found in all the clonally expanded CD8^+^ cells as seen in classic T-LGL leukemia. One of these patients first displayed lymphoproliferative symptoms at the age of 8 while the other presented at the age of 31. Other cases have also demonstrated either T-cell hyperplasia of the bone marrow or marginal T-LGL clone in the peripheral blood, but not meeting criteria for T-LGL leukemia (8). These reports suggest a link between T-cell proliferation and deficiency of ADA2. Furthermore, a case report describes a patient with DADA2 presenting with clinical features mimicking autoimmune lymphoproliferative syndrome, including persistent fever, generalized lymphadenopathy, and hepatosplenomegaly (9). Bone marrow biopsy showed 90% hypercellularity without other abnormalities. A cervical lymph node biopsy showed florid follicular lymphoid cortical hyperplasia. After diagnosis of DADA2 was made, the patient had a good response to TNF inhibition. Our case and others demonstrate the need to both monitor for lymphoproliferative disorders in known DADA2 patients and to consider DADA2 in differential diagnosis of patients presenting with early-onset immune dysregulations.

There are limited detailed descriptions of the gastrointestinal involvement in DADA2. Gastrointestinal manifestations may include hepatomegaly and/or splenomegaly, portal hypertension, hepatoportal sclerosis, and ischemic bowel disease. Hepatic involvement is variable and with a reported prevalence up to 67% in one cohort (1, 10). Most commonly the liver histology in DADA2 demonstrates NRH. Interestingly, prior studies have demonstrated liver pathology in diseases marked by hypogammaglobulinemia. Of particular interest, 5–12% of patients with CVID can develop NRH (11, 12). Three particular subtypes of NRH have been described in CVID including: (a) non-progressive NRH, (b) NRH that progresses to portal hypertension, and (c) NRH associated with autoimmune hepatitis-like syndrome (NRH/AIH) (11). The later subtype (NRH/AIH) is particularly resistant to immunosuppressive therapies and progresses rapidly to hepatic dysfunction, portal hypertension, and jaundice. The time course of Patient 1 is very similar to what has been presented in NRH/AIH associated with CVID, which is suggestive that DADA2 may have similar subgroups. It also emphasizes that TNF inhibitors may be ineffective in preventing progressive liver decline if initiated late in the disease course. Although, our case emphasizes the need to monitor for liver involvement closely in DADA2, it is unclear whether current therapies are effective in inhibiting the progression to cirrhosis in patients with advanced disease.

The two siblings with DADA2 presented here, diagnosed at the age of 40 and 43, respectively, represent some of the oldest patients reported to date, as there are very few reports of patients diagnosed as adults (2, 10, 13, 14). Cases of DADA2 diagnosed later in life provide a unique opportunity to examine the natural history of the disease and demonstrate the potential for these patients to develop life-threatening sequela if untreated. Furthermore, these cases highlight a need for greater awareness about DADA2 among neurologists when evaluating adults with unexplained neurological features, including recurrent and/or early-onset stroke and inflammation; and among rheumatologists, when evaluating cases of suspected PAN. Disease-associated mutations in the *ADA2* gene may be associated with significant phenotypic variability in individuals with the same genotype, emphasizing a possible role for other modifying gene alleles and/or environmental factors in the pathogenesis of this disease.

## Ethics Statement

All subjects gave written informed consent in accordance with the Declaration of Helsinki.

## Author Contributions

JS and IA developed the manuscript. ND and IA did the genetic testing and serum ADA2 levels. SG, an immunologist who followed the patients, provided expertise. HJ and DK did autopsy on patient and provided autopsy pictures. AO and PG gathered patient data and provided expertise. All the authors approved of the final manuscript.

## Conflict of Interest Statement

The authors declare that the research was conducted in the absence of any commercial or financial relationships that could be construed as a potential conflict of interest.
